# Lack of association between TDP-43 pathology and tau mis-splicing in Alzheimer's disease

**DOI:** 10.1016/j.neurobiolaging.2015.09.022

**Published:** 2016-01

**Authors:** Michael Niblock, Tibor Hortobágyi, Claire Troakes, Safa Al-Sarraj, Carl Spickett, Rebecca Jones, Christopher E. Shaw, Jean-Marc Gallo

**Affiliations:** a Department of Basic and Clinical Neuroscience, Maurice Wohl Clinical Neuroscience Institute, Institute of Psychiatry, Psychology and Neuroscience, King's College London, London, United Kingdom; b London Neurodegenerative Disease Brain Bank, Institute of Psychiatry, Psychology and Neuroscience, King's College London, London, United Kingdom; c Division of Psychiatry, Faculty of Brain Sciences, University College London, London, United Kingdom

**Keywords:** TDP-43, Tau, Splicing, Alzheimer's disease, Tauopathy

## Abstract

A proportion of Alzheimer's disease cases displays inclusions of the RNA-binding protein, TDP-43. Considering the pathogenic role of tau mis-splicing, we compared tau isoform expression between Alzheimer's disease cases with or without TDP-43 inclusions. The average ratio of tau isoforms containing or lacking exon 10 (4R/3R ratio) or the total level of tau mRNA was not significantly different between cases with or without TDP-43 pathology in any of the brain regions examined. Although TDP-43 functions may be affected, TDP-43 does not critically regulate expression or splicing of tau in Alzheimer's disease suggesting that TDP-43 contributes to Alzheimer's disease through mechanisms independent of tau.

## 1 Introduction

Alzheimer's disease (AD) is characterized by Aβ and tau pathologies in affected brain areas. In addition, 20%–30% of AD cases display cytoplasmic inclusions of transactive response DNA-binding protein of 43 kDa (TDP-43). The presence of TDP-43 inclusions in the hippocampus and entorhinal cortex correlates with neuronal loss in late onset dementia and, furthermore, a strong correlation exists between the presence of TDP-43 pathology and specific clinical features of AD, especially impaired cognition and amnesia (Davidson et al., 2011). TDP-43 is a predominantly nuclear protein that regulates RNA splicing and stability of multiple targets; therefore, mislocalization and aggregation of TDP-43 could contribute to pathogenesis in a subgroup of AD cases by affecting directly or indirectly the level of expression or splicing of specific targets. As mis-splicing of tau exon 10 (E10) can be pathogenic (Niblock and Gallo, 2012), we compared tau expression and splicing in affected brain regions in AD patients with or without TDP-43 pathology to establish a possible contribution of abnormal TDP-43 activity in AD through tau splicing.

## 2 Methods

Frozen brain tissue was obtained from individuals free from neurological disease and individuals with AD and controls. Cases were assessed for TDP-43 pathology by immunocytochemistry. RNA was extracted using the Qiagen RNeasy/lipid kit (Qiagen). Tau E10 splicing was analyzed by end-point reverse transcription polymerase chain reaction using a forward primer 5′-labelled with an infrared fluorescent dye, yielding a signal independent of the length of the individual products. Gels were imaged, and polymerase chain reaction products were quantified using an Odyssey Infrared Imaging System (LI-COR Biosciences) (Supplementary Fig. 2A). Statistical analysis was carried out using one-way ANOVA and Student *t* test.

## 3 Results

We analyzed tau splicing in a cohort of 14 AD cases with (ADTDP+) and 15 AD cases without (ADTDP−) TDP-43 inclusions and 15 age-matched nondemented healthy controls (Supplementary Table 1). The amygdala is consistently affected by tau pathology in AD and, in cases with TDP-43 pathology, the amygdala consistently contains TDP-43 inclusions. In the amygdala, the average molar ratio of isoforms containing or lacking E10 (4R/3R ratio) ranged between 0.7 and 1.2 in control cases. Individually, the ADTDP+ and ADTDP− groups showed a borderline significant increase in the 4R/3R ratio compared to controls (Supplementary Fig. 2B). However, the average 4R/3R ratios were not significantly different between the ADTDP+ and ADTDP− groups.

## 4 Discussion

Mutations in the *TARDBP* gene, encoding TDP-43, have been linked to familial forms of amyotrophic lateral sclerosis (Sreedharan et al., 2008), thus arguing for a pathogenic role of TDP-43. Tau pre-mRNA has not been found to be a direct target of TDP-43 (Tollervey et al., 2011). However, TDP-43 is part of an RNA regulatory complex that includes fused in sarcoma (FUS). FUS binds to tau pre-mRNA, and FUS downregulation promotes E10 inclusion in tau mRNA in rodents (Orozco et al., 2012). Whether tau mis-splicing contributes to sporadic AD has been a matter of debate (Niblock and Gallo, 2012). Considering the pathological importance of tau splicing and the role of TDP-43 as a splicing regulator, it was important to determine whether a correlation existed between the presence of TDP-43 pathology in sporadic AD cases and the 4R/3R ratio in affected brain areas. Levels of E10 inclusion were not significantly different between AD cases with or without TDP-43 pathology; however, we found a borderline significant increase in 4R tau in the combined AD cases compared to controls. The results could have been confounded by parameters inherent to the use of postmortem diseased brain tissue such as changes in cell population resulting from cell loss and gliosis. In the present study, we compared individual transcripts between 2 disease subgroups that would have a similar extent of cell population change and, in addition, we found no difference in tau mRNA levels between AD and control cases which reflects the quality of the material selected. In conclusion, although TDP-43 RNA regulatory functions may be affected in a subgroup of Alzheimer's disease cases, TDP-43 does not critically regulate expression or splicing of tau suggesting that TDP-43 contributes to clinical features of Alzheimer's disease through mechanisms independent of tau. Considering the role of TDP-43 as a regulator of RNA processing and its importance in dementia, large scale genome-wide studies are required to determine the significance of TDP-43 pathology in Alzheimer's disease.

## Disclosure statement

The authors declare that they have no conflict of interest.
