# Supplementary material for: Lack of association between TDP-43 pathology and tau mis-splicing in Alzheimer's disease
Source: Neurobiol Aging. 2016 Jan;37:45–6. doi: 10.1016/j.neurobiolaging.2015.09.022 (PMC4706155; doi:10.1016/j.neurobiolaging.2015.09.022)
Supplement: Supplementary Material [file mmc1.pdf]

Supplementary material

Extended Introduction

TDP-43 inclusions in Alzheimer's disease (AD), when present, are mainly cytoplasmic, but are also found, but much more rarely, in the nucleus (Chang et al., 2001; Amador-Ortiz et al., 2007; Davidson et al., 2011). The hippocampus and entorhinal cortex, when TDP-43 positive, usually show very high TDP inclusion loads (Hu et al., 2008; Arai et al., 2009). To some extent, the presence of TDP-43 inclusions correlates with some pathological or clinical aspects of AD (Davidson et al., 2011; Josephs et al., 2014; Keage et al., 2014; Josephs et al., 2015).

TDP-43 is a predominantly nuclear RNA- and DNA-binding protein that is the principal component of ubiquitinated inclusions in amyotrophic lateral sclerosis (ALS) and tau-negative frontotemporal lobar degeneration with TDP-43-positive inclusions (FTLD-TDP) (Neumann et al., 2006). TDP-43 regulates several aspects of DNA and RNA metabolism, including splicing and stability of multiple targets (Polymenidou et al., 2011; Tollervey et al., 2011; Xiao et al., 2011). A transcript that could be affected directly or indirectly by mislocalization and aggregation of TDP-43 in AD is tau pre-mRNA.

Tau is the main component of paired helical filaments. Tau is encoded by the *MAPT* gene that produces six isoforms by alternative splicing of exons 2, 3, and 10 [reviewed in (Gallo et al., 2007; Caillet-Boudin et al., 2015)]. Exon 10 (E10) mis-splicing can be pathogenic as a number of cases of frontotemporal dementia with parkinsonism linked to chromosome 17 (FTDP-17-*MAPT*) are caused by dominant mutations in the *MAPT* gene affecting *cis*-acting elements regulating E10 splicing and resulting in an excess of E10-containing tau isoforms (Hutton et al., 1998; Spillantini et al., 1998). Increased E10 inclusion

is likely to have a functional significance as it results in an excess of tau isoforms containing four microtubule-binding repeats (4R tau) in the C-terminus of the protein.

Previous comparative analyses of tau splicing by quantitative or semi-quantitative RT-PCR have, in most cases, failed to show differences in 4R/3R ratios between AD patients and controls (Chambers et al., 1999; Boutajangout et al., 2004; Umeda et al., 2004; Connell et al., 2005; Ingelsson et al., 2006). Some studies, however, have reported increases in 4R tau transcripts in sporadic AD (Yasojima et al., 1999; Ginsberg et al., 2006; Glatz et al., 2006; Conrad et al., 2007). Importantly, the *MAPT* H1 haplotype, a risk factor for AD, promotes E10 inclusion in tau mRNA (Myers et al., 2005; Caffrey et al., 2006).

Although tau is not a direct target of TDP-43 (Polymenidou et al., 2011; Tollervey et al., 2011; Xiao et al., 2011), abnormal RNA processing activity of TDP-43 in AD cases with TDP-43 pathology could indirectly alter tau splicing and contribute to disease. However we found no significant difference in tau splicing between AD cases with or without TDP-43 pathology, suggesting that TDP-43 contributes to clinical features of Alzheimer's disease through mechanisms independent of tau. These results are consistent with the notion that TDP-43 and tau pathologies are independent of each other in frontotemporal lobar degeneration with tau pathology (FTLD-tau) (Robinson et al., 2014).

Extended Methods

Brain tissue

Frozen brain tissue was obtained from individuals free from neurological disease and individuals with AD (Braak stages V- VI) from the MRC London Neurodegenerative Diseases Brain Bank (Institute of Psychiatry, Psychology and Neuroscience, King's College London, UK) (Supplementary Table 1). Consents for autopsy, neuropathological assessment, and research, including genetic studies, were obtained from all subjects in accordance with

local and national research ethics guidelines. All cases were genotyped and confirmed to have no known mutations in *MAPT*, *APP*, *PS1* or *PS2.* Samples from the amygdala, the hippocampus, the frontal and the temporal cortex, and the cerebellum were dissected and tissue was stored at -70°C until analysis. Control cases had no clinical history of a neurodegenerative condition, in particular dementia. FTDP-17 samples, all with the 10+16 mutation, had been identified at the Institute of Neurology, Queen Square, London.

All AD cases were neuropathologically confirmed as classical AD and a Braak stage assigned according to established neuropathological criteria. Control status was confirmed by the absence of plaques or tangles and by the absence of AD-type changes other than those consistent with aging. TDP-43 status was determined using an antibody recognizing TDP-43 phosphorylated at Ser409/410 that specifically detects ubiquitin-positive TDP-43 inclusions and does not detect soluble nuclear TDP-43 (CosmoBio).

All cases were genotyped for their *MAPT* haplotype. DNA was extracted from cerebellum samples from control and AD cases with the phenol/chloroform method. The H2 haplotype was identified by a 238 bp deletion in intron 9 not present in the H1 haplotype (Baker et al., 1999).

RNA extraction and reverse transcription

100 mg of frozen brain tissue was homogenized in matrix lysing-D tubes (MP Biomedicals) in conjunction with the Fastprep sample preparation system (MP Biomedicals). Total RNA was extracted from homogenized samples using the Qiagen RNeasy/lipid kit (Qiagen) according to the manufacturer's protocol. RNA was eluted in 40 μl of RNase-free water and treated with DNase using a DNA-free kit (Applied Biosystems) according to the manufacturer's instructions. RNA concentration was determined using a Nanodrop spectrophotometer (Thermo Scientific). RNA integrity number (RIN) was measured with the Agilent RNA 6000 Pico kit using an Agilent 2100 BioAnalyzer (Agilent Technologies, Santa

Clara, CA, USA). RNA was reverse transcribed using oligo(dT) and reagents from the Taqman RT Kit (Life Technologies). PCR was performed with GoTaq polymerase reagents (Promega).

Quantitative RT-PCR

Real-time qRT-PCR was performed in 384-well plates with 20 ng template cDNA. For total tau RNA analysis, a forward primer spanning exons 11 and 12 and a reverse primer spanning exons 12 and 13 were used: forward: 5'-CCATCATAAACCAGGAGGTGGCC-3' reverse: 5'-GGTCAGCTTGTGGGTTTCAATCTT-3'. This primer pair produced a single PCR product and its identity was confirmed by sequencing. The two most stable reference genes for each brain region were selected using GeNorm^{PLUS} kit and GeNorm^{PLUS} software (Primerdesign, UK). qPCR was performed on an Applied Biosystems 7900HT Fast Real-Time PCR System using SYBR Green (Roche Diagnostics) and the following cycling parameters: 95°C for 10 min, 40 cycles of 15 s at 94°C, 30 s at 60°C and a final step of 15 s at 72°C. Samples were run in duplicate and the average cycle threshold (Ct) was calculated for target and two reference genes. Total levels of *MAPT* expression from each brain region were calculated relative to the geometric mean of two housekeeping genes. Statistical analysis was carried out using one way ANOVA and Student's *t* test.

RT-PCR analysis of tau splicing

Tau E10 splicing was analyzed by end-point RT-PCR. Primers were designed on the basis of the GenBank cDNA sequences using the primer designing tool, Primer3, and synthesized commercially (MWG Eurofins). The forward primer was modified by conjugating the DY682 fluorophore at the 5' end. PCR was performed using the following primers and cycling parameters: forward: 5'-DY682-CTGAAGCACCAGCCAGGAGG-3', reverse: 5'-AGCCACATCGCTCAGACAC-3', 95°C for 5 min, 28 cycles of 2 min at 94°C, 30 s at 60°C, 20 s at 72°C, and a final step of 10 min at 72°C; *GAPDH*: forward: 5'-

GCCCAATACGACCAAATCC-3’, reverse: 5’-AGCCACATCGCTCAGACAC-3’; 95°C for 5 min, 28 cycles of 30 s at 94°C, 30 s at 62°C, 1 min at 72°C, and a final step of 10 min at 72°C. PCR products were separated by electrophoresis in 2% (w/v) agarose gels. Gels were imaged and PCR products were quantified using an Odyssey Infrared Imaging System® (LI-COR Biosciences). Statistical analysis was carried out using one way ANOVA and Student’s *t*-test. Comparisons were made between individual ADTDP+ and ADTDP- groups and control and between ADTDP+ and ADTDP- groups.

Protein analysis

Brain samples were homogenized with a mechanical homogenizer (Ultra Turrax® T8, Werke GmbH & Co., Germany) in 50 mM Tris/HCl, pH 7.4, 150 mM NaCl, 1 mM PMSF and a protease inhibitor cocktail (Complete® EDTA-free, Roche). Samples were centrifuged at 12,000 g for 20 min at 4ºC and the supernatant containing both soluble and insoluble tau was collected (Greenberg and Davies, 1990). For the extraction of insoluble tau, 1% (w/v) sarkosyl was added to supernatant from the low speed centrifugation and the samples were centrifuged at 100,000 g for 1 h at 21ºC. The resulting pellet contained detergent-insoluble tau and was solubilized with 8 M guanidine and 2% (v/v) β-mercaptoethanol. Low speed centrifugation fraction and guanidine-solubilized tau were dialyzed against 50 mM Tris/HCl, pH 8.0 and 0.1 mM EDTA and dephosphorylated with λ phosphatase (New England Biolabs) (Hanger et al., 2002). Protein concentration was measured using the BCA assay (Pierce). Proteins were separated in 10% SDS-PAGE gels along with a tau ladder made of all recombinant tau isoforms (Sigma) and transferred to a 0.45 μm pore size nitrocellulose membrane. Non-specific protein binding sites were blocked with 5% (w/v) skimmed milk in PBS + 0.1% (v/v) Tween-20 (PBST). Membranes were incubated overnight at 4°C with a rabbit polyclonal antibody to the C-terminus of human tau (aa 243-441; DakoCytomation) or with a GAPDH antibody for normalization of protein loadings. Immunoreactivity was

detected with an IRDye800-conjugated anti-rabbit antibody and membranes were imaged using an Odyssey Infrared Imaging System® (LI-COR Biosciences). Individual tau isoforms were identified as 3R and 4R-type by comparison with the recombinant tau ladder. Tau isoforms were quantified and their relative proportions calculated. Statistical analysis was carried out using one way ANOVA and Student's *t*-test.

Additional Results

Tau mRNA levels in the presence or absence of TDP-43 pathology

Total levels of tau mRNA were compared by real-time qRT-PCR between AD cases with (ADTDP+) or without (ADTDP-) TDP-43 pathology and control cases in affected brain regions (i.e. the amygdala, the hippocampus and the frontal and the temporal cortex) as well as in the cerebellum (Supplementary Fig. 1). The level of tau mRNA was similar between control and disease conditions and no significant differences were found between the ADTDP+ and ADTDP- groups or between each individual groups and controls in any of the affected brain region examined. Similarly, there were no significant differences in total tau RNA expression between ADTDP+, ADTDP- and control groups in the cerebellum, an area typically not affected in AD.

Tau E10 splicing in the presence or absence of TDP-43 pathology

The amygdala is of particular interest as it is consistently affected by tau pathology in AD and, in cases with TDP-43 pathology, the amygdala consistently contains TDP-43 inclusions (Amador-Ortiz et al., 2007; Arai et al., 2009). In the amygdala, the average molar ratio of isoforms containing or lacking E10 (4R/3R ratio) in control cases ranged between 0.7 and 1.2 (Supplementary Fig. 2). A ~3-4 4R/3R ratio was measured in FTDP-17 cases with the +16 *MAPT* mutation. These values are consistent with previous reports using other methods (Hutton et al., 1998; Boutajangout et al., 2004; Connell et al., 2005; Ingelsson et al., 2006).

Individually, the ADTDP+ and ADTDP- groups showed a borderline significant increase in the 4R/3R ratio compared to controls, however, the average 4R/3R ratios were not significantly different between the ADTDP+ and ADTDP- groups in any of the brain regions examined (Supplementary Fig. 3). Sequencing of the *MAPT* gene around E10 in cases with a high 4R/3R ratio did not detect *de novo* or somatic mutations within exonic or intronic E10 splicing regulatory elements showed that could have resulted in enhanced E10 inclusion. The H1 *MAPT* haplotype is a risk factor for AD and is associated with increased E10 inclusion (Myers et al., 2005; Caffrey et al., 2006). The high level of E10 inclusion in some cases is unlikely to be related to the H1 haplotype as no predominance of a particular *MAPT* haplotype was found in control or AD cases in the cohort analyzed (Supplementary Table 1). However this analysis does not rule out an association of high E10 expression with other SNPs in the H1 clade (Myers et al., 2005; Myers et al., 2007).

Analysis of tau protein

Tau isoform ratios were compared at the protein level between ADTDP+ and ADTDP- cases in the amygdala. All isoforms were detected in sarkosyl-insoluble fractions, that contain aggregated tau (Supplementary Fig. 4A) as well as in soluble fractions (Supplementary Fig. 4B). Of note, insoluble tau was under the level of detection in control brains (Supplementary Fig. 4A). Consistent with increased E10 inclusion, cases with the *MAPT* +16 mutation showed high levels of 4R tau isoforms compared to AD cases in the sarkosyl-insoluble fractions (Supplementary Fig. 4A). The 4R/3R average ratios in the sarkosyl-insoluble fractions of ADTDP+ and ADTDP- cases (0.71 and 0.75, respectively; Supplementary Fig. 4C) were not significantly different. The tau isoform pattern in soluble fractions was similar between AD cases and control brains, irrespective of the TDP-43 status (Supplementary Fig. 4B).

References

Amador-Ortiz, C., Lin W.L., Ahmed Z., Personett D., Davies P., Duara R., Graff-Radford N.R., Hutton M.L., Dickson D.W., 2007. TDP-43 immunoreactivity in hippocampal sclerosis and Alzheimer's disease. Ann. Neurol. 61, 435-445.

Arai, T., Mackenzie I.R., Hasegawa M., Nonoka T., Niizato K., Tsuchiya K., Iritani S., Onaya M., Akiyama H., 2009. Phosphorylated TDP-43 in Alzheimer's disease and dementia with Lewy bodies. Acta Neuropathol. 117, 125-136.

Baker, M., Litvan I., Houlden H., Adamson J., Dickson D., Perez-Tur J., Hardy J., Lynch T., Bigio E., Hutton M., 1999. Association of an extended haplotype in the tau gene with progressive supranuclear palsy. Hum. Mol. Genet. 8, 711-715.

Boutajangout, A., Boom A., Leroy K., Brion J.P., 2004. Expression of tau mRNA and soluble tau isoforms in affected and non-affected brain areas in Alzheimer's disease. FEBS Lett. 576, 183-189.

Caffrey, T.M., Joachim C., Paracchini S., Esiri M.M., Wade-Martins R., 2006. Haplotype-specific expression of exon 10 at the human *MAPT* locus. Hum. Mol. Genet. 15, 3529-3537.

Caillet-Boudin, M.L., Buee L., Sergeant N., Lefebvre B., 2015. Regulation of human *MAPT* gene expression. Mol. Neurodegener. 10, 28.

Chambers, C.B., Lee J.M., Troncoso J.C., Reich S., Muma N.A., 1999. Overexpression of four-repeat tau mRNA isoforms in progressive supranuclear palsy but not in Alzheimer's disease. Ann. Neurol. 46, 325-332.

Chang, J.G., Hsieh-Li H.M., Jong Y.J., Wang N.M., Tsai C.H., Li H., 2001. Treatment of spinal muscular atrophy by sodium butyrate. Proc. Natl. Acad. Sci. USA 98, 9808-9813.

Connell, J.W., Rodriguez-Martin T., Gibb G.M., Khan N.M., Grierson A.J., Hanger D.P., Revesz T., Anderton B.H., Gallo J.-M., 2005. Quantitative analysis of tau isoform transcripts in sporadic tauopathies. Mol. Brain Res. 137, 104-109.

Conrad, C., Zhu J., Schoenfeld D., Fang Z., Ingelsson M., Stamm S., Church G., Hyman B.T., 2007. Single molecule profiling of tau gene expression in Alzheimer's disease. J. Neurochem. 103, 1228-1236.

Davidson, Y.S., Raby S., Foulds P.G., Robinson A., Thompson J.C., Sikkink S., Yusuf I., Amin H., DuPlessis D., Troakes C., Al-Sarraj S., Sloan C., Esiri M.M., Prasher V.P., Allsop D., Neary D., Pickering-Brown S.M., Snowden J.S., Mann D.M., 2011. TDP-43 pathological changes in early onset familial and sporadic Alzheimer's disease, late onset Alzheimer's disease and Down's syndrome: association with age, hippocampal sclerosis and clinical phenotype. Acta Neuropathol. 122, 703-713.

Gallo, J.-M., Noble W., Rodriguez-Martin T., 2007. RNA and protein-dependent mechanisms in tauopathies: consequences for therapeutic strategies. Cell. Mol. Life Sci. 64, 1701-1714.

Ginsberg, S.D., Che S., Counts S.E., Mufson E.J., 2006. Shift in the ratio of three-repeat tau and four-repeat tau mRNAs in individual cholinergic basal forebrain neurons in mild cognitive impairment and Alzheimer's disease. J. Neurochem. 96, 1401-1408.

Glatz, D.C., Rujescu D., Tang Y., Berendt F.J., Hartmann A.M., Faltraco F., Rosenberg C., Hulette C., Jellinger K., Hampel H., Riederer P., Moller H.J., Andreadis A., Henkel K., Stamm S., 2006. The alternative splicing of tau exon 10 and its regulatory proteins CLK2 and TRA2-BETA1 changes in sporadic Alzheimer's disease. J. Neurochem. 96, 635-644.

Greenberg, S.G., Davies P., 1990. A preparation of Alzheimer paired helical filaments that displays distinct tau proteins by polyacrylamide gel electrophoresis. Proc. Natl. Acad. Sci. USA 87, 5827-5831.

Hanger, D.P., Gibb G.M., de Silva R., Boutajangout A., Brion J.P., Revesz T., Lees A.J., Anderton B.H., 2002. The complex relationship between soluble and insoluble tau in tauopathies revealed by efficient dephosphorylation and specific antibodies. FEBS Lett. 531, 538-542.

Hu, W.T., Josephs K.A., Knopman D.S., Boeve B.F., Dickson D.W., Petersen R.C., Parisi J.E., 2008. Temporal lobar predominance of TDP-43 neuronal cytoplasmic inclusions in Alzheimer disease. Acta Neuropathol. 116, 215-220.

Hutton, M., Lendon C.L., Rizzu P., Baker M., Froelich S., Houlden H.H., Pickering Brown S., Chakraverty S., Isaacs A., Grover A., Hackett J., Adamson J., Lincoln S., Dickson D., Davies P., Petersen R.C., Stevena M., De Graaff E., Wauters E., Van Baren J., Hillebrand M., Joosse M., Kwon J.M., Nowotny P., Che L.K., Norton J., Morris J.C., Reed L.A., Trojanowski J., Basun H., Lannfelt L., Neystat M., Fahn S., Dark F., Tannenberg T., Dodd P.R., Hayward N., Kwok J.B.J., Schofield P.R., Andreadis A., Snowden J., Craufurd D., Neary D., Owen F., Costra B.A., Hardy J., Goate A., Van Swieten J., Mann D., Lynch T., Heutink P., 1998. Association of missense and 5'-splice-site mutations in tau with the inherited dementia FTDP-17. Nature 393, 702-704.

Ingelsson, M., Ramasamy K., Cantuti-Castelvetri I., Skoglund L., Matsui T., Orne J., Kowa H., Raju S., Vanderburg C.R., Augustinack J.C., de Silva R., Lees A.J., Lannfelt L., Growdon J.H., Frosch M.P., Standaert D.G., Irizarry M.C., Hyman B.T., 2006. No alteration in tau exon 10 alternative splicing in tangle-bearing neurons of the Alzheimer's disease brain. Acta Neuropathol. 112, 439-449.

Josephs, K.A., Whitwell J.L., Tosakulwong N., Weigand S.D., Murray M.E., Liesinger A.M., Petrucelli L., Senjem M.L., Ivnik R.J., Parisi J.E., Petersen R.C., Dickson D.W., 2015. TAR DNA-binding protein 43 and pathological subtype of Alzheimer's disease impact clinical features. Ann. Neurol.

Josephs, K.A., Whitwell J.L., Weigand S.D., Murray M.E., Tosakulwong N., Liesinger A.M., Petrucelli L., Senjem M.L., Knopman D.S., Boeve B.F., Ivnik R.J., Smith G.E., Jack C.R., Jr., Parisi J.E., Petersen R.C., Dickson D.W., 2014. TDP-43 is a key player in the clinical features associated with Alzheimer's disease. Acta Neuropathol. 127, 811-824.

Keage, H.A., Hunter S., Matthews F.E., Ince P.G., Hodges J., Hokkanen S., Highley J.R., Dening T., Brayne C., 2014. TDP-43 in the population: prevalence and associations with dementia and age. J. Alzheimers Dis. 42, 641-650.

Myers, A.J., Kaleem M., Marlowe L., Pittman A.M., Lees A.J., Fung H.C., Duckworth J., Leung D., Gibson A., Morris C.M., de Silva R., Hardy J., 2005. The H1c haplotype at the *MAPT* locus is associated with Alzheimer's disease. Hum. Mol. Genet. 14, 2399-2404.

Myers, A.J., Pittman A.M., Zhao A.S., Rohrer K., Kaleem M., Marlowe L., Lees A., Leung D., McKeith I.G., Perry R.H., Morris C.M., Trojanowski J.Q., Clark C., Karlawish J., Arnold S., Forman M.S., Van D.V., de Silva R., Hardy J., 2007. The *MAPT* H1c risk haplotype is associated with increased expression of tau and especially of 4 repeat containing transcripts. Neurobiol. Dis. 25, 561-570.

Neumann, M., Sampathu D.M., Kwong L.K., Truax A.C., Micsenyi M.C., Chou T.T., Bruce J., Schuck T., Grossman M., Clark C.M., McCluskey L.F., Miller B.L., Masliah E., Mackenzie I.R., Feldman H., Feiden W., Kretzschmar H.A., Trojanowski J.Q., Lee V.M., 2006. Ubiquitinated TDP-43 in frontotemporal lobar degeneration and amyotrophic lateral sclerosis. Science 314, 130-133.

Polymenidou, M., Lagier-Tourenne C., Hutt K.R., Huelga S.C., Moran J., Liang T.Y., Ling S.C., Sun E., Wancewicz E., Mazur C., Kordasiewicz H., Sedaghat Y., Donohue J.P., Shiue L., Bennett C.F., Yeo G.W., Cleveland D.W., 2011. Long pre-mRNA depletion and RNA missplicing contribute to neuronal vulnerability from loss of TDP-43. Nat. Neurosci. 14, 459-468.

Robinson, A.C., Thompson J.C., Weedon L., Rollinson S., Pickering-Brown S., Snowden J.S., Davidson Y.S., Mann D.M., 2014. No interaction between tau and TDP-43 pathologies in either Frontotemporal Lobar Degeneration or Motor Neurone Disease. Neuropath. Appl. Neurobiol. 40, 844-854.

Spillantini, M.G., Murrell J.R., Goedert M., Farlow M.R., Klug A., Ghetti B., 1998. Mutation in the tau gene in familial multiple system tauopathy with presenile dementia. Proc. Natl. Acad. Sci. USA 95, 7737-7741.

Tollervey, J.R., Curk T., Rogelj B., Briese M., Cereda M., Kayikci M., Konig J., Hortobagyi T., Nishimura A.L., Zupunski V., Patani R., Chandran S., Rot G., Zupan B., Shaw C.E., Ule J., 2011. Characterizing the RNA targets and position-dependent splicing regulation by TDP-43. Nat. Neurosci. 14, 452-458.

Umeda, Y., Taniguchi S., Arima K., Piao Y.S., Takahashi H., Iwatsubo T., Mann D., Hasegawa M., 2004. Alterations in human tau transcripts correlate with those of neurofilament in sporadic tauopathies. Neurosci. Lett. 359, 151-154.

Xiao, S., Sanelli T., Dib S., Sheps D., Findlater J., Bilbao J., Keith J., Zinman L., Rogaeva E., Robertson J., 2011. RNA targets of TDP-43 identified by UV-CLIP are deregulated in ALS. Mol. Cell. Neurosci. 47, 167-180.

Yasojima, K., McGeer E.G., McGeer P.L., 1999. Tangled areas of Alzheimer brain have upregulated levels of exon 10 containing tau mRNA. Brain Res. 831, 301-305.

Supplementary Table 1
Demographic, clinical, pathological and genetic data from the cases used.

Case	PM diagnosis	TDP-43 status	Haplotype	Age	Sex	PMD
1	Control	TDP-43-	H1/H1	78	M	9.5
2	Control	TDP-43-	H2/H2	80	F	3
3	Control	TDP-43-	H1/H2	66	M	52
4	Control	TDP-43-	H1/H1	38	F	43
5	Control	TDP-43-	H1/H1	78	M	24
6	Control	TDP-43-	nd	18	M	24.5
7	Control	TDP-43-	H1/H2	81	M	18
8	Control	TDP-43-	H1/H2	90	F	50
9	Control	TDP-43-	H1/H1	92	F	48
10	Control	TDP-43-	H1/H1	84	F	35
11	Control	TDP-43-	H1/H2	80	M	60
12	Control	TDP-43-	H1/H1	97	M	44
13	Control	TDP-43-	H1/H1	84	F	34
14	Control	TDP-43-	H1/H1	89	F	41
15	Control	TDP-43-	nd	92	F	23
16	AD	TDP-43+	H1/H2	74	M	69
17	AD	TDP-43+	H1/H2	73	M	24.5
18	AD	TDP-43+	H1/H1	86	F	14
19	AD	TDP-43+	H1/H1	85	M	16
20	AD	TDP-43+	H1/H1	89	M	62
21	AD	TDP-43+	H1/H1	97	M	16.5
22	AD	TDP-43+	H1/H1	85	F	10
23	AD	TDP-43+	H1/H1	86	M	45
24	AD	TDP-43+	H1/H2	77	M	9.5
25	AD	TDP-43+	H1/H1	69	M	120
26	AD	TDP-43+	H1/H2	88	F	33
27	AD	TDP-43+	H1/H2	90	F	70
28	AD	TDP-43+	H1/H1	68	F	10.5
29	AD	TDP-43+	H1/H1	83	F	76
30	AD	TDP-43-	H1/H1	96	F	39
31	AD	TDP-43-	H1/H1	92	F	4
32	AD	TDP-43-	H1/H2	80	F	12.5
33	AD	TDP-43-	H1/H2	84	M	73
34	AD	TDP-43-	H1/H1	69	F	16.3
35	AD	TDP-43-	H1/H1	96	F	19
36	AD	TDP-43-	H1/H1	84	F	24
37	AD	TDP-43-	H1/H1	97	F	12
38	AD	TDP-43-	H1/H1	80	M	41
39	AD	TDP-43-	H1/H1	91	F	28.5
40	AD	TDP-43-	H1/H1	81	M	74
41	AD	TDP-43-	H1/H1	71	M	32
42	AD	TDP-43-	H1/H1	82	F	69
43	AD	TDP-43-	H2/H2	86	M	25.5
44	AD	TDP-43-	H1/H1	91	F	28.5
45	FTDP-17	TDP-43-	nd	60	M	3
46	FTDP-17	TDP-43-	nd	63	F	22
47	FTDP-17	TDP-43-	nd	67	M	35
48	FTDP-17	TDP-43-	nd	71	M	5
49	FTDP-17	TDP-43	nd	52	F	72

Key: PMD: post mortem delay in hours.

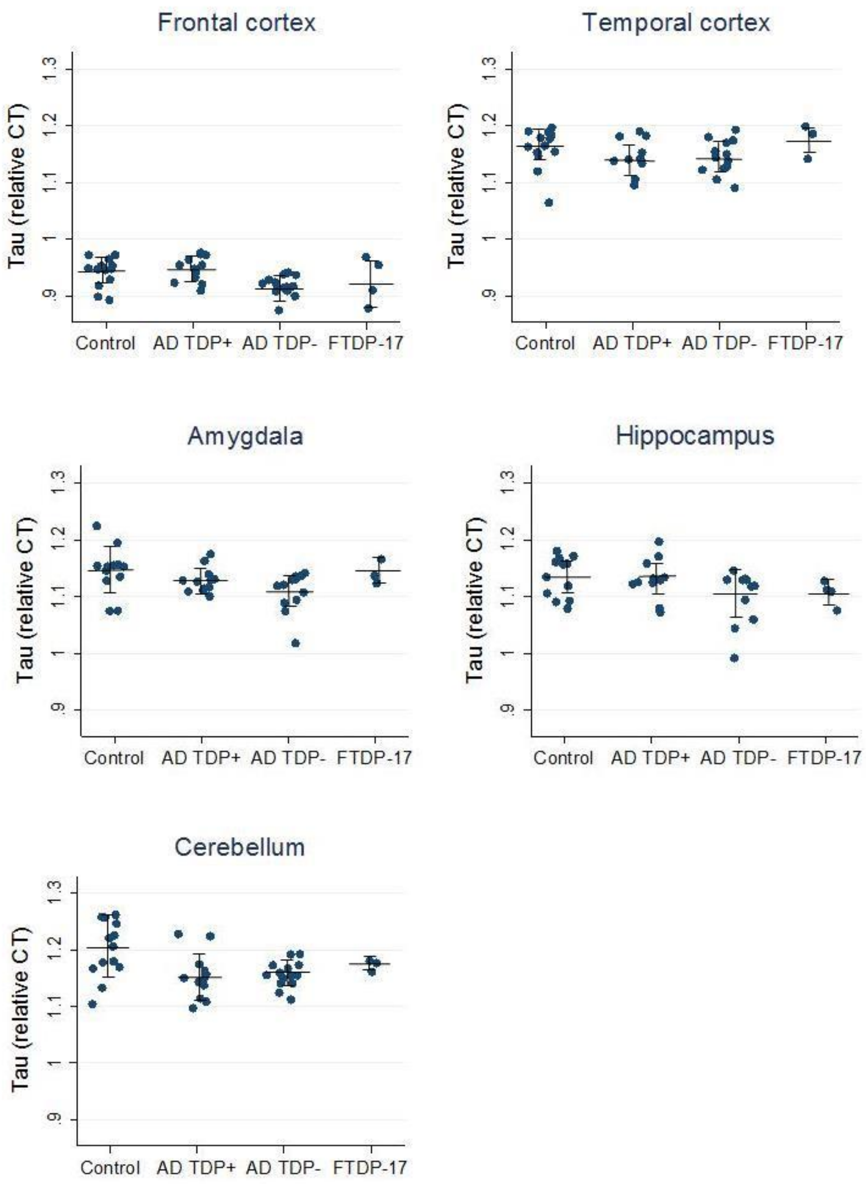

Supplementary Fig. 1. Comparison of tau mRNA expression across brain regions in TDP-43 positive and TDP-43 negative Alzheimer's disease cases. Total levels of tau mRNA in the amygdala, the hippocampus, the frontal and the temporal cortex and the cerebellum were compared between ADTDP+, ADTDP-, FTDP-17 and control cases by real-time qRT-PCR. Tau mRNA levels showed no significant differences between control and disease conditions or between the ADTDP+ and ADTDP- groups in any of the brain regions examined. Each data point represents an individual case.

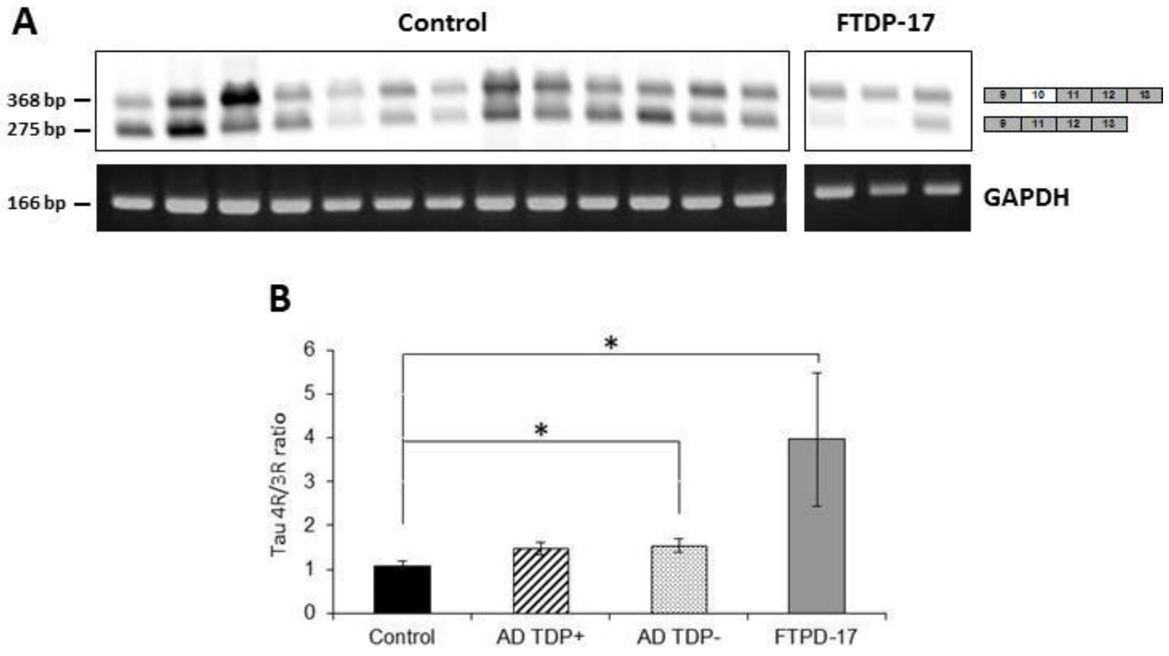

Supplementary Fig. 2. (A) Analysis of tau 4R/3R ratio by end-point RT-PCR in the amygdala using a DY682-labelled forward primer specific for exon 9 and a reverse primer specific for exon 13. (B) Comparison of tau E10 splicing in the amygdala of TDP-43-positive and TDP-43-negative Alzheimer's disease cases as well as in FTDP-17 cases with the +16 *MAPT* mutation. Data represent mean 4R/3R ratio ± SEM. n=14 (ADTDP+), 15 (ADTDP-), 15 (control), *P<0.05. 4R/3R ratios showed no significant differences between the ADTDP+ and ADTDP- groups.

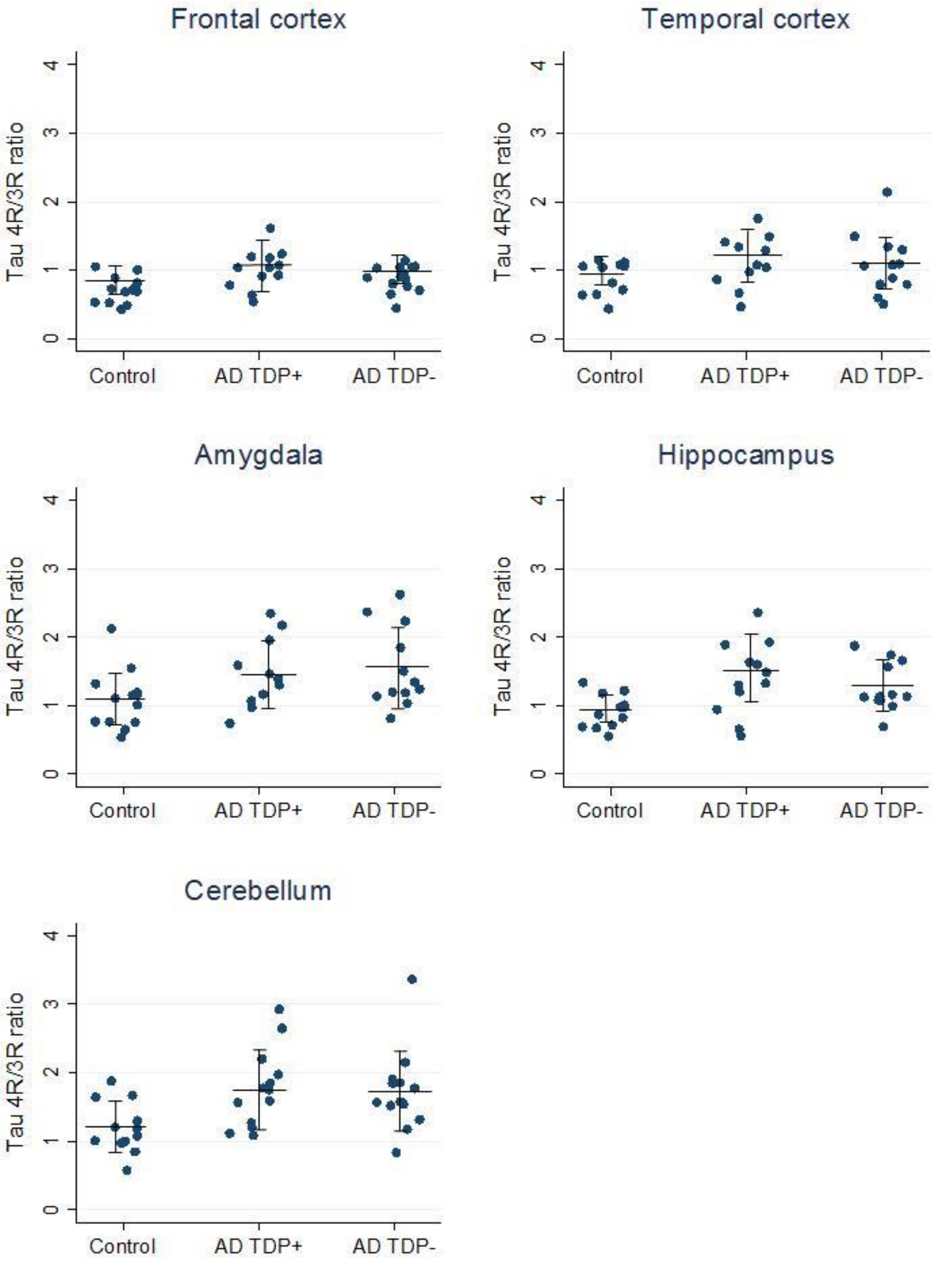

Supplementary Fig. 3. Comparison of tau E10 splicing across brain regions in TDP-43 positive and TDP-43 negative Alzheimer's disease cases. Tau E10 splicing in the amygdala, the hippocampus, the frontal and the temporal cortex and the cerebellum were compared between ADTDP+, ADTDP- and control cases as in Fig. 2. 4R/3R ratios showed no significant differences between the ADTDP+ and ADTDP- groups in any of the brain regions examined. Each data point represents an individual case.

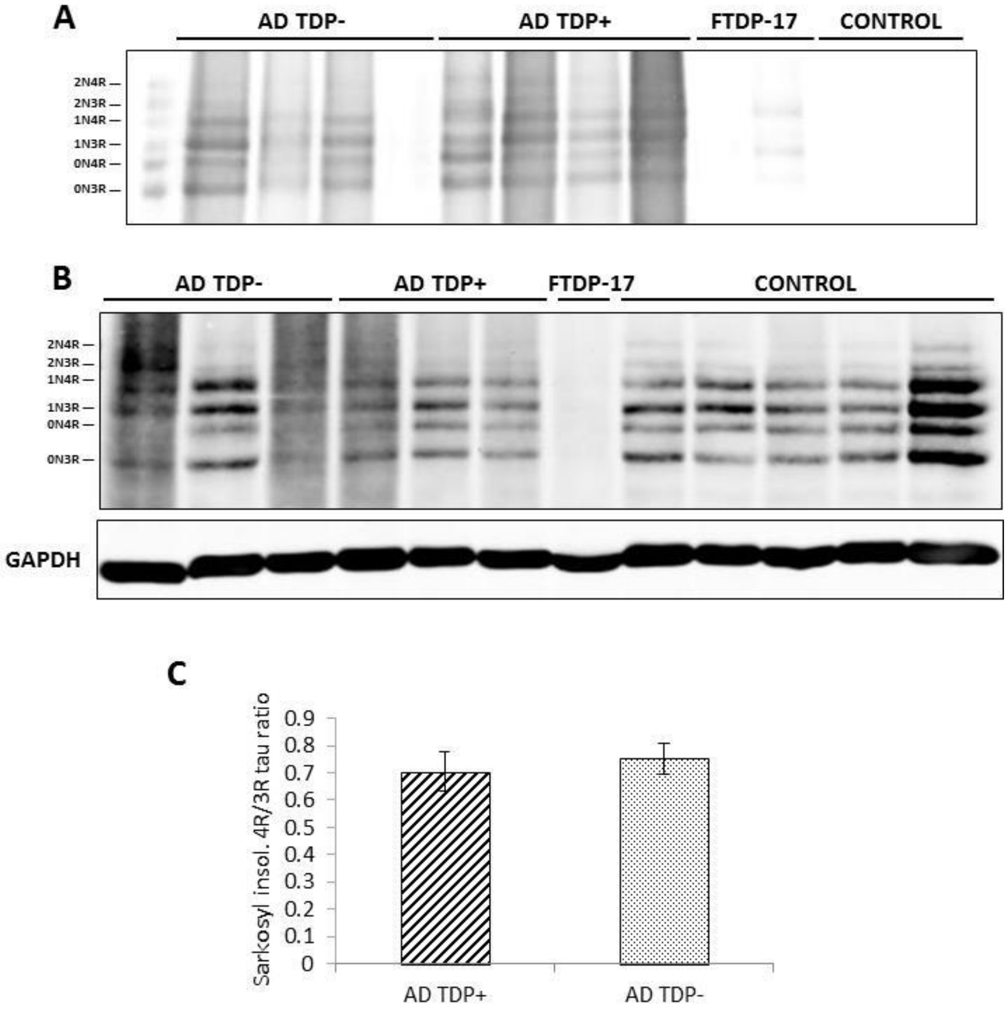

Supplementary Fig. 4. Comparison of soluble and insoluble tau protein in the amygdala of TDP-43 positive and TDP-43 negative Alzheimer's disease cases, FTDP-17 cases with the +16 *MAPT* mutation, and control cases. GAPDH was used as a loading control. (A) Representative pattern of sarkosyl-insoluble dephosphorylated tau. (B) Representative pattern of soluble dephosphorylated tau. (C) Comparison of tau isoform ratios in sarkosyl-insoluble fractions in the amygdala of ADTDP+ and ADTDP- cases. The average 4R/3R protein ratio was not significantly different between the ADTDP+ and ADTDP- groups. Data represent mean 4R/3R ratio ± SEM. n=9 (AD TDP+), 11 (ADTDP-).
